# HIV knowledge and associated factors among young Ethiopians: application of multilevel order logistic regression using the 2016 EDHS

**DOI:** 10.1186/s12879-020-05436-2

**Published:** 2020-09-29

**Authors:** Teshome Kabeta Dadi, Merga Belina Feyasa, Mamo Nigatu Gebre

**Affiliations:** 1 Department of Epidemiology, Jimma University, Institute of Health, Faculty of Public Health, Jimma, Ethiopia; 2 Department of Statistics, Addis Ababa University, College of Natural & Computational Sciences, Addis Ababa, Ethiopia

**Keywords:** HIV knowledge, Youths, EDHS, Multilevel, Order logistic regression

## Abstract

**Background:**

Human Immunodeficiency virus continues to be a major global health problem infecting 75 million and killing 32 million people since the beginning of the epidemic. It badly hit Sub Saharan Africa than any country in the world and youths are sharing the greatest burden. The study aims to assess the level of HIV-knowledge and its determinants among Ethiopian youths using the 2016 Ethiopia Demographic and Health Survey data.

**Methods:**

A nationally representative 2016 Ethiopian Demographic and Health Survey data were used. A total of 10,903 youths comprising 6401 females and 4502 males were included in the study. Descriptive statistics and multilevel order logistic regression were used and confidence interval was used to declare statistical significance in the final model.

**Results:**

The mean age and SD of youths included in this study was 19.10 (±2.82). Among Ethiopian youths, 20.92% (95% CI: 18.91, 23.09%) had low knowledge of HIV whereas, 48.76% (95% CI: 47.12, 50.41%) and 30.31% (95% CI: 28.51, 32.18%) of them had moderate and comprehensive HIV knowledge respectively. Being male, access to TV and radio, ever tested for HIV/AIDS, owning a mobile telephone, and attending primary school and above compared to non-attendants were associated with having higher HIV knowledge. But, dwelling in rural Ethiopia, being in the Protestant and Muslim religious groups as compared to those of Orthodox followers and being in married groups were associated with having lower HIV knowledge. Approximately, 12% of the variation in knowledge of HIV was due to regions.

**Conclusion:**

Only one-third of Ethiopian youths have deep insight into the disease, whereas, nearly one-fifth of them have lower HIV-knowledge. There is a significant disparity in HIV-related knowledge among Ethiopian youths living in different regions. Rural residents, less educated, female, and married youths have less knowledge of HIV as compared to their counterparts. Youths who do not have a mobile phone, who lack health insurance coverage, and who have limited access to media have less knowledge about HIV. Therefore, the due focus should be given to the aforementioned factors to minimize the disparities between regions and to enhance Ethiopian youths’ HIV-knowledge.

## Background

HIV continues to be a major global health issue. Since the beginning of the epidemic, 75 million people have been infected with HIV and about 32 million people have died of it [1–3]. The Joint United Nations Program on HIV (UNAIDS) and other partners had launched an ambitious plan of diagnosing 90% of all HIV-positive persons, providing ART for 90% of those diagnosed, and achieving viral suppression for 90% of those treated by 2020 which was believed to enable the world to end the AIDS epidemic by 2030 [4]. Even though aggressive responses were made to HIV/AIDS by different stakeholders, the 2019 reports from USAID and WHO showed that 37.9 million people were still living with HIV/AIDs at the end of 2018. The same reports had witnessed that 1.7 million people were newly infected with the disease and 770,000 people died from the disease-related causes in the same year [2, 3]. The global annual number of HIV new infections had declined from 2.1 million in 2010 to 1.7 million in 2018, which left the world far off the 2020 target to have fewer than 500,000 new infections. The globally annual number of deaths from HIV/AIDS-related illnesses among People living with HIV (PLWH) had also fallen from a peak of 1.7 million in 2004 to 770,000 in 2018. However, reaching the 2020 milestone of fewer than 500,000 deaths will still require further declines of 135,000 deaths per year [3, 5]. Sub-Saharan Africa (SSA) disproportionately carries a higher burden of HIV accounting for more than 70% of the global burden of infection. Of the estimated 6000 new infections that occur globally each day, about two-thirds are in SSA [6]. East and Southern Africa are especially the home to the largest number of PLWH where 20.6 million were PLWH and 800,000 were newly infected in 2018 [7]. A geospatial analysis of national survey data collected from SSA showed that national-level HIV prevalence for young adults ranged from 2.2% in Tanzania to 7.7% in Mozambique [8]. According to a 2016 WHO report, an estimated number of 710,000 PLWH in Ethiopia where the estimated adult HIV prevalence was 1.1% [9]. However, the number of PLWH was decreased to 690,000 in 2018 when an estimated number of 23,000 people were newly infected [5].

Youths are people between the ages of 15 and 24 years [10]. Globally, 3.9 million youths were living with HIV and there were 590,000 new infections among global youths in 2017. Approximately 1600 youth acquire HIV every day, and one young person dies of AIDS-related illness every 10 min worldwide [11]. According to 2020 WHO estimates, over 30% of all new HIV infections globally are estimated to occur among youths aged 15 to 25 years [12]. Globally, new HIV infections among young women were reduced by 25% between 2010 and 2018, but 6000 adolescent girls and young women become infected with HIV every week [5]. Adolescent girls and young women have up to eightfold higher rates of HIV infection compared to their male peers [6]. Of the 37,832 new HIV diagnoses in the US in 2018, 21% were among youth [13]. In sub-Saharan Africa, adolescent girls and young women aged 15–24 years are particularly vulnerable to HIV infection. Even though they accounted for only one-tenth of the total population residing in the region, adolescent girls and young women in SSA accounted for 25% of all new HIV infections globally in 2017. Eighty percent of all HIV infections occurring among adolescents in SSA occur among youth girls aged 15–19 years [14]. There was 290,000 new HIV infection among youths in Eastern and Southern Africa in 2017 which was the highest of all HIV incidences that occurred among youths across the globe in the same year [11]. In Ethiopia, 690, 000 people were living with HIV in 2018, whereas, 23, 000 people were newly infected and 11,000 people died from an AIDS-related illness in the same year. Women are disproportionally affected by HIV in Ethiopia: of the 650, 000 adults living with HIV, 410, 000 (63.08%) were women. At the end of 2018, new HIV infections among young women aged 15–24 years were more than double of those among young men: 5800 young women were newly infected compared to 2000 new infection among young men. In Ethiopia, in 2018, only 79% of people living with HIV knew their HIV status and only 65% of people living with HIV were on treatment [15].

The potential risks for young people for becoming newly infected with HIV are closely linked with the age of sexual initiation. Therefore, abstinence from sexual intercourse and delayed initiation of sexual behavior are among the central aims of HIV prevention efforts for young people [12]. Studies done in Iran and Ethiopia showed that only a few proportions of young people do have comprehensive HIV-knowledge [16–18]. Exposure to mass media, being from a rich family, ever HIV VCT uptake, receiving education on HIV, being male, socioeconomic status, and employment status were significantly associated with HIV-knowledge among youths [17–20].

The 2016 Ethiopian demographic and health survey (EDHS) report depicted that only 39% of youth males and 24% of youth females had comprehensive HIV-knowledge [21]. Even though 61% of youth males and nearly three-fourth of youth females did not have comprehensive HIV-knowledge, to the knowledge of the authors, proximate determinants of comprehensive HIV-knowledge among youths in the country are not well studied. Therefore, the current study is aimed to study the determinants of HIV-knowledge among Ethiopian youths using the 2016 EDHS data.

## Methods

### Data sources

The Central Statistical Agency (CSA) has conducted EDHS since 2000 by the request of the Ministry of Health (MoH). The 2016 EDHS was the fourth DHS survey conducted in Ethiopia that comes every 5 years by getting technical assistance from ICF through the DHS Program, which is funded by the United States Agency for International Development (USAID) and offers support and technical assistance for the implementation of population and health surveys in different countries worldwide. Further analyses have been done on this data by securing permission of working further analyses from the MEASUREDHS program. Variables used to measure HIV knowledge by the six domains and other variables that have potential associations were identified, extracted, processed, and re-analyzed.

### Sampling techniques and study population for the parent study

The sampling frame used for the 2016 EDHS was the Ethiopia Population and Housing Census (PHC) conducted in 2007 by the CSA consisting of a complete list of 84,915 households used for the 2007 PHC. An EA is a geographic area covering an average of 181 households. The selection of the sample was in two stages, from each region which was stratified into urban and rural areas. In the first stage, a total of 645 EAs, 202 in urban, and 443 in rural, was selected with probability proportional to EA size and with independent selection in each sampling stratum. In the second stage of selection, a fixed number of 28 households per cluster were selected with an equal probability systematic selection from the updated household list. A total of 15,683 women aged 15–49 and 12,688 men aged 15–59 who were either permanent residents of the selected households or visitors who stayed in the household the night before the survey were interviewed in the EDHS 2016 [22]. For the current study, 6401 young females aged 15–24 and 4502 young males aged 15–24 were screened from totally interviewed sexually active males and females for further analysis.

### Measurements and operational definitions

The levels of HIV knowledge among youths were computed from the scoring of six questions asked regarding their understanding of HIV. These domains were whether the respondents have knowledge about the possibility of reducing the risk of getting HIV by always using condoms during sex and whether they know about the possibility of reducing the risk of getting HIV by having only one sex partner, who has no other partners. The other four domains were related to the misconceptions about HIV: a healthy-looking person can have HIV, can get HIV by witchcraft or supernatural means, can get HIV from mosquito bites, and can get HIV by sharing food with a person who has AIDS [23]. On the other hand, types of occupation in DHS studies are commonly too many and intensive grouping was done according to the previously published study [24].

Based on the reviewed literatures and availability of relevant variables in DHS studies, region, sex, age, religion, wealth status, marital status, occupation, residence, sex of household head, education, owning of cellphone, ever use of the internet, frequency of using the internet, frequency of reading newspaper in a month, frequency of listening to the radio in a week, frequency of watching television in a week, ever test for HIV and health insurance coverage status variables were considered for the current study.

### Operational definitions regarding levels of HIV knowledge and marriage

#### Low HIV knowledge

Low HIV knowledge if the scores of the six domains of HIV knowledge measurement are summed to 0 to 3.

#### Moderate HIV knowledge

Referred to be a moderate level of knowledge of HIV if the sum of the response to the six domains is between 4 and 5.

#### Comprehensive HIV knowledge

The level of knowledge of HIV is said to be comprehensive if the sum of the response to the six domains of HIV knowledge scores is 6.

#### Never in a union

Refers to youths who have never been married in any form of marriage.

### Data analysis

Data management and analyses were carried out using Stata 14.2 statistical software. To assess regional variation of HIV-knowledge and identify factors associated with the outcome of interest the multilevel ordinal logistic regression model was employed as the nature of the data follows a hierarchy. The appropriate statistical method that can capture variability due to the application of staged sampling (two-stage stratified cluster sampling) is multilevel analysis [25]. When the variance of the residual errors is correlated between individual observations as a result of these nested structures, single ordinal logistic regression is inappropriate [26].

In classical regression, estimates of varying effects can be noisy, especially when there are few observations per group; multilevel modeling allows us to estimate interactions to the extent supported by the data. The proportion of total variation in the response variable that is accounted for the between-group variation is captured by Intra Class Correlation (ICC) [26]. The regions where the participants were dwelling were considered as a clustering variable. Moreover, all predictors are at level 1, and studying the effect of the clustering variable was of interest.

The results for descriptive analysis were done by using weights provided in EDHS 2016 data and adjusted for multilevel analysis as per the recommendation by Adam [27]. The model fitted by using the adjusted weights had lower AIC = 16,514.5, and BIC = 16,593.94 compared to results from unadjusted weights where AIC = 18,834.06 and BIC = 18,906.28. The final fitted model with significant variables, lower AIC and BIC was considered for further discussion, and results are portrayed in Table 1.

**Table 1:** Result of multilevel ordinal logistic regression on HIV knowledge among youths in Ethiopia, EDHS 2016

Predictors	Odds Ratio	Robust Std. Err.	z	***P*** > z	[95% Conf. Int.]	
**Residence**						
Urban (Ref.)						
Rural	0.7195	0.0675	−3.5100	0.0000	0.5987	0.8647
**Educational**						
No Education (Ref.)						
Primary	2.1839	0.1865	9.1500	0.0000	1.8474	2.5817
Secondary	3.3879	0.3827	10.8000	0.0000	2.7150	4.2274
Higher	3.8515	0.5410	9.6000	0.0000	2.9245	5.0722
**Owns mobile**						
No (Ref.)						
Yes	1.2757	0.0576	5.3900	0.0000	1.1676	1.3938
**HIV test**						
No (Ref.)						
Yes	1.5186	0.1142	5.5500	0.0000	1.3105	1.7599
**Freq. of listening radio**						
Not at all (Ref.)						
< 1 a week	1.2421	0.0581	4.6400	0.0000	1.1333	1.3612
> = a week	1.2760	0.1159	2.6800	0.0070	1.0679	1.5247
**Freq. of watching TV**						
Not at all (Ref.)						
< 1 a week	1.1958	0.0832	2.5700	0.0100	1.0433	1.3706
> = a week	1.4241	0.1206	4.1800	0.0000	1.2064	1.6813
**Health insurance**						
No (Ref.)						
Yes	1.4485	0.1317	4.0700	0.0000	1.2120	1.7311
**Respondent’s sex**						
Female (Ref.)						
Male	1.8500	0.1050	10.8400	0.0000	1.6553	2.0675
**Religion**						
Orthodox (Ref.)						
Protestant	0.7143	0.0922	−2.6100	0.0090	0.5547	0.9198
Muslim	0.7454	0.0998	−2.1900	0.0280	0.5734	0.9691
Other	0.6670	0.1166	−2.3200	0.0210	0.4735	0.9396
**Marital Status**						
Never in a union (Ref.)						
Married	0.7918	0.0493	−3.7500	0.0000	0.7009	0.8945
Other	0.9572	0.1094	−0.3800	0.7020	0.7652	1.1975
/cut1^a^	−0.2286	0.1383	−1.6500	0.0980	−0.4996	0.0424
/cut2	2.4053	0.1398	17.2100	0.0000	2.1314	2.6792
**Region**						
var.(_cons)	0.1659	0.1035			0.0488	0.5637

^a^_cut1 – This is the estimated cut point on the latent variable used to differentiate *low* **level of knowledge of HIV** from *moderate* and *comprehensive* **level of knowledge of HIV** when values of the predictor variables are evaluated at zero.

_cut2 – This is the estimated cut point on the latent variable used to differentiate *low* and *moderate* **level of knowledge of HIV** from *comprehensive* **level of knowledge of HIV** when values of the predictor variables are evaluated at zero.

## Results

### Characteristics of the participants

**Fig. 1:**
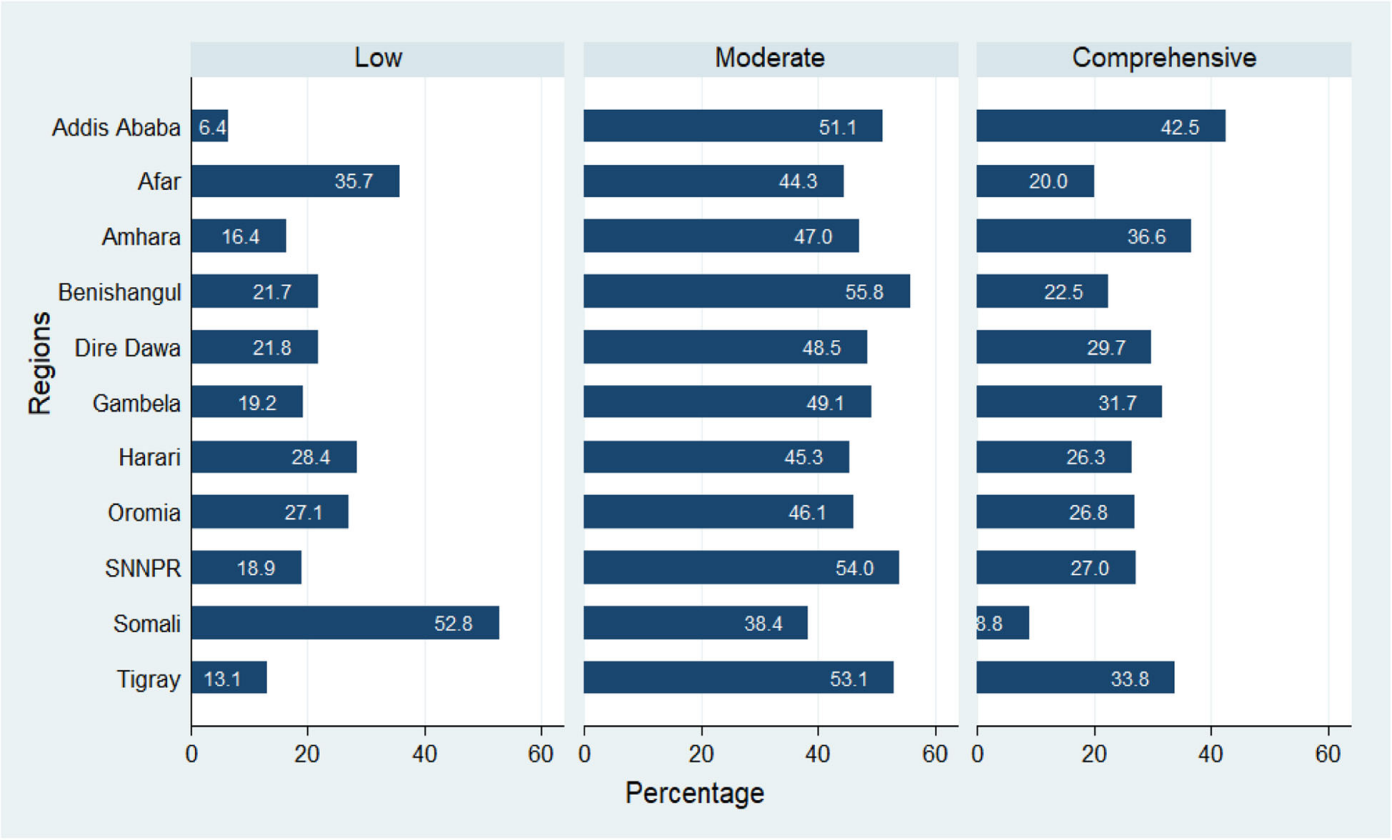
Levels of HIV knowledge prevalence among youths by regions in Ethiopia, 2016 EDHS

A total of 10,903 adolescents with a mean age of 19.10 (±2.82) years were included in this study. The majority (57.3%) of the youths attended primary school, and almost 17.0% didn’t attend school. Most (78.0%) of the youths were rural dwellers. About 77.0% of youths belong to male-headed households. Almost two in five of the youths didn’t work at all in the last 12 months before the survey. Nearly 34% of the respondents were engaged in agricultural works. Most of the youths (43.9%) were Orthodox Christians followed by Muslims (30.4%). Most of the youths (69.7%) were never been in a union; whereas one-fourth of the youths were married. Fifty-eight percent of the youths were female and almost two in five of the youths own mobile telephones. The majority (67%) of the youths belong to at least a middle-class family (Table 2). The prevalence of levels of knowledge of HIV among youths in Ethiopia by regions is presented in Fig. 1.

**Table 2:** Socio-demographic and economic characteristics of young Ethiopians, EDHS 2016

Items	Category			Level of knowledge of HIV					
		Total		Low		Moderate		Comprehensive	
		N	%	N	%	N	%	N	%
**Education**	No education	1773	16.7	632	44.3	582	40.7	214	15.0
	Primary	6076	57.3	1307	22.5	2893	49.8	1607	27.7
	Secondary	2095	19.8	135	6.5	1083	52.0	866	41.5
	Higher	654	6.2	12	1.8	305	46.7	336	51.5
**Residence**	Urban	2335	22.0	200	8.7	1123	48.7	984	42.7
	Rural	8263	78.0	1887	24.6	3740	48.8	2039	26.6
**Sex of HH head**	Male	8104	76.5	1633	21.5	3735	49.1	2237	29.4
	Female	2494	23.5	454	19.2	1128	47.7	786	33.2
**Occupation**	Not working	4262	40.2	1052	26.8	1912	48.7	961	24.5
	Agricultural	3572	33.7	672	19.8	1595	47.0	1128	33.2
	Professional	253	2.4	12	4.9	128	50.9	111	44.3
	Trade/Sales	1014	9.6	151	15.3	517	52.1	322	32.6
	Elementary	968	9.1	135	14.7	470	51.2	313	34.1
	Others	528	5.0	62	12.7	242	49.2	187	38.1
**Wealth Status**	Poor	3450	32.6	943	30.6	1443	46.8	697	22.6
	Middle	1960	18.5	439	24.0	911	49.8	478	26.2
	Rich	5187	49.0	705	13.9	2509	49.6	1848	36.5
**Religion**	Orthodox	4647	43.9	656	14.6	2210	49.0	1643	36.4
	Protestant	2500	23.6	494	20.7	1247	52.2	647	27.1
	Muslim	3222	30.4	863	30.1	1315	45.9	689	24.0
	Other	229	2.2	74	35.2	91	43.6	44	21.3
**Marital Status**	Never in union	7388	69.7	1194	17.1	3425	49.0	2375	34.0
	Married	2684	25.3	779	31.3	1207	48.3	505	20.3
	Other	525	5.0	114	23.3	230	47.3	143	29.4
**Own’s Mobile**	No	6264	59.1	1632	28.5	2699	47.2	1393	24.3
	Yes	4334	40.9	455	10.7	2164	50.9	1630	38.4
**Respondent’s sex**	Female	6143	58.0	1526	26.7	2820	49.3	1375	24.0
	Male	4455	42.0	560	13.2	2043	48.1	1648	38.8

### Level of knowledge of HIV among young Ethiopians

The current study shows that 20.92% (95% CI: 18.91, 23.09%) of Ethiopian youths have low knowledge on HIV, whereas, 48.76% (95% CI: 47.12, 50.41%) and 30.31% (95% CI: 28.51, 32.18%) of them had moderate and comprehensive knowledge on HIV respectively (Table 3).

**Table 3:** Level of knowledge of HIV among young Ethiopians, EDHS 2016

			Total		Level of HIV Knowledge					
					Low		Moderate		Comprehensive	
			N	%	N	%	N	%	N	%
**Knowledge of HIV**					2086	20.9	4863	48.8	3023	30.3
**Measurements of HIV knowledge by domain**	**Can people reduce their chance of getting HIV by using a condom every time they have sex?**	**No**	1912	21.0	840	44.0	1072	56.0	0	0.0
		**Yes**	7167	79.0	601	8.4	3572	49.0	3024	42.0
	**Can people reduce their chance of getting HIV by having just one uninfected sex partner who has no other sex partners?**	**No**	1625	20.7	753	46.3	872	54.0	0	0.0
		**Yes**	7819	79.3	917	1.7	3879	49.6	3023	38.6
	**Is it possible for a healthy-looking person to have HIV?**	**No**	2400	25.3	1005	41.0	1395	58.1	0	0.0
		**Yes**	7074	74.7	726	10.2	3325	47.0	3023	43.0
	**Can people get HIV because of witchcraft or other supernatural means?**	**No**	8532	89.5	1164	14.0	4345	51.0	3023	35.0
		**Yes**	1001	10.5	627	62.0	374	37.0	0	0.0
	**Can people get HIV from mosquito bites?**	**No**	6363	70.7	558	9.0	2782	44.0	3023	48.0
		**Yes**	2640	29.3	974	37.0	1666	63.0	0	0.0
	**Can people get HIV by sharing food with a person who** **has HIV?**	**No**	8561	80.0	1071	13.0	4467	52.0	3023	35.0
		**Yes**	2131	20.0	838	69.0	375	31.0	0	0

### Result of multilevel ordinal logistic regression

In all the following presentation of results as well as discussions on odds ratio refers to the adjusted odds ratio. The residence has a statistically significant association with the level of knowledge of HIV. The odds of having a comprehensive knowledge of HIV, instead of low to moderate knowledge of HIV, for rural dwellers, was lower by approximately 30%; AOR = 0.7195 with 95% CI [0.5987, 0.8647]. Education has a statistically significant association with the level of knowledge of HIV, with AOR = 2.1839 95% CI [1.8474, 2.5817] for primary, AOR = 3.3879 95% CI [2.7150, 4.2274] for secondary and AOR = 3.8515 95% CI [2.9245, 5.0722] for high-schoolers respectively. The odds of having a comprehensive knowledge of HIV instead of low to moderate knowledge on HIV was AOR = 1.2757 and 95% CI [1.1676, 1.3938] for mobile telephone owners compared to non-owners. Youths those who have already tested for HIV/AIDS were more likely to have a comprehensive knowledge of HIV compared to those who were never tested for HIV, AOR = 1.5186 with a 95% CI [1.3105, 1.7599]. Access to media like TV and radio were also found to have a statistically significant association with the level of knowledge on HIV; those with a higher frequency of access were more likely to have compressive knowledge on HIV.

For insured youths, the odds of having comprehensive knowledge of HIV was AOR = 1.4485 with a 95% CI [1.2120, 1.7311]. The odds of having comprehensive knowledge instead of low to moderate, for young males, was AOR = 1.8500 with a 95% CI [1.6553, 2.0675] as compared to females. Protestants AOR = 0.7143 with a 95% CI [0.5547, 0.9198] and Muslim, AOR = 0.7454 with a 95% CI [0.5734, 0.9691], were less likely to have a comprehensive knowledge on HIV compared to their Orthodox counterparts. Marital status was also significantly associated with the level of knowledge on HIV of the youths, AOR = 0.7918 with a 95% CI [0.7009, 0.8945]. A significant amount, approximately 12% with CI [4.97, 26.80%] of the variation of prevalence of level of knowledge of HIV was accounted for by the regions.

## Discussion

Results show that 20.92% of youths aged 15–24 have low knowledge of HIV, whereas, 48.76 and 30.31% of them had a moderate and comprehensive level of knowledge on HIV respectively. In the EDHS report, the percentage of females and males having a comprehensive knowledge of HIV was 24 and 39% respectively [22].

Rural dwellers are less likely to have a comprehensive knowledge of HIV, instead of low to moderate knowledge of HIV. This is consistent with the findings from Nigeria and Bangladesh where rural residents were less likely to have HIV-knowledge compared to the urban residents [28, 29]. This could be due to differences in access to mass media between urban and rural youths which play a very vital role in disseminating HIV-related educations and awareness creations. This study and other studies [18, 29–39] depicted that exposure to mass media is associated with acquiring comprehensive HIV-knowledge.

Unambiguously education is expected to increase knowledge in almost all dimensions. The result of this study was not against this statement. The odds of having comprehensive HIV-knowledge among youths who completed primary school compared to those with no education were more than two-fold. Youths who completed secondary and above were more likely to have a comprehensive knowledge of HIV than low to a moderate HIV-knowledge compared to those with no education. This finding is also consistent with the findings from Nigeria and Bangladesh which were done using DHS data where youths with better educational attainment had better knowledge of HIV [28, 29]. This could be due to the integration of HIV-related education in academic curricula and the information grasping and analytical capacity of educated people.

For mobile owners compared to non-owners, the odds of having a comprehensive knowledge of HIV instead of low to moderate knowledge of HIV was higher by more than 27%. This finding is similar to the study done on adolescents from the United States and Botswana, where adolescents who discussed peer pressure and connectedness with mobile phones and social media had a general knowledge of Sexually Transmitted Infections (STIs) and HIV [38]. On the other hand, studies from Ghana, Uganda, and the United States elucidated that mobile phone usage among youths is useful for delivering HIV related information and HIV prevention [36, 37, 39]. This puts mobile phones at the heart of other strategies used to disseminate HIV/AIDS-related educations and awareness creations.

Generally, it is unquestionable that differences in getting information can result in differences in the level of knowledge that one has and this study affirms this fact. Not only access but also the frequency of accessing information matters and this is reflected in the current study. The odds of having comprehensive HIV-knowledge instead of having low to moderate for those watching TV less than once a week and at least once a week was higher by 20 and 42% than those not watching at all respectively. Similarly, the odds of having comprehensive HIV-knowledge instead of low to moderate among those listening to the radio for less than once a week and at least once a week was higher by 24 and 27% than those not listening at all respectively. This result is concordant with the findings from 27 SSA countries and India where people who frequently watch television and frequently listen to the radio had more comprehensive HIV-knowledge than people who do not [34, 35]. Several studies [18, 29–33] had also witnessed that exposure to mass media had an impact on acquiring comprehensive HIV-knowledge, due to the HIV-related educations and information conveyed through mass media. This might be explained by differences in exposure to HIV related information between those who do have frequent access to mass media and those who do not have as mass media are potential sources of information.

Despite the infancy of the implementation during the data collection period, the coverage of health insurance has a significant association with HIV-knowledge. Results show that the odds of having comprehensive HIV-knowledge instead of having a low to moderate among the insured youths were 45% higher than those with no health insurance coverage. This could be due to the reason that youths who do have health insurance might have more access to health facilities than youths who are not covered under health insurance. Youths who have more access to health facilities, in turn, might have more health-related knowledge than those who do not have access.

The sex of the respondents has a significant association with the level of HIV-knowledge among youths. The odds of having comprehensive HIV-knowledge instead of low to moderate level among males were 85% higher than females. This finding contradicts the result described by studies conducted in Nigeria, where females are more likely to have HIV-knowledge than that of the males [40]. Additionally, the study in Sub Saharan Africa (SSA) uncovered that females have a positive association with having better knowledge than males [41]. Contrary to this, sex did not affect having HIV knowledge in the study done among university students in the United Arab Emirates (UAE) [42]. The current study is similar to the assessment of comprehensive HIV/AIDS knowledge levels among in-school adolescents in eastern Ethiopia and the study done in Nigeria using DHS data, where females were less likely to have comprehensive HIV/AIDS knowledge compared to males [18, 28]. Different studies have shown that there are gender disparities in academic achievement in different countries. In more civilized and industrialized countries females have more academic achievement than males [43–46]. But in developing countries like Ethiopia, females have less access and less academic achievement than males [47]. So, one of the possible reasons for discrepancies in HIV-knowledge among males and females in studies done in different areas and the current study may be due to differences in access to education and academic achievement. On the other hand, it is evidenced in the current study and other studies, that access to media increases HIV-related knowledge. Studies have shown that females are more economically disadvantaged and have less access to media than males in many countries [48, 49]. Therefore, gender disparities in HIV-related knowledge between males and females may also be due to differences in accessing media.

It is expected that religion affects many things, it can make differences in knowing HIV knowledge and this expectation is supported by this study. The odds of having comprehensive HIV knowledge among Orthodox Christians instead of having low to moderate was by 29, 25, and 33% higher than Protestant Christians, Muslims, and other belief followers respectively. The current study reaffirms the study conducted in China in that following different religions has contributed to making a variation of HIV knowledge [50]. The possible reason for this could be differences in conveying HIV-related information between different religious organizations. Consistent condom use was one of the domains for measuring HIV knowledge but the ministers of any religion confidently speak about this prevention method and it is clear that the followers were likely to not consider the method as means of prevention and hence the disparity can be linked to the effect of views reflected while preaching in Churches and Mosques.

The result of this study uncovered that the marital status of the youths has a significant association with the level of HIV-knowledge. The odds of having comprehensive HIV-knowledge instead of having low to moderate for married youths compared to never-married were lower by about 21%, controlling for the effect of the other predictors in the model. In a study conducted among university students in UAE, marital status didn’t make a difference on level HIV-knowledge [42] but the current finding agrees with the study conducted in Vietnam in that those who live with their spouses were more likely to have knowledge of HIV than singles [51]. This probably is because of the different age groups included in either of the studies and also it could be due to cultural and norm differences across the countries in which the studies were conducted.

Results from multilevel regression show that there was significant variability due to residing in different regions of Ethiopia. The variation on HIV-knowledge due to regions accounted for 12% of the variability and this could be attributed to the difference in the quality of health systems across the regions of the country. As depicted in Fig. 1, the distribution of the prevalence of moderate knowledge on HIV among youths in Ethiopia is more or less similar across regions. On the other hand, there were differences across regions on both low and comprehensive knowledge of HIV.

### Strength of the study

The results yielded from the current study are more valid than the results from any other prior small-scale studies done in the country as the sampling techniques, the data collection process, and the data processing and management of the 2016 EDHS, which was used for the current study, were very rigorous and to the standard. Besides, the study is of sufficient power as the 2016 EDHS, which we used for the current analysis included a large sample size. On the other hand, weighting of the data was done prior to the analyses to minimize any kind of bias that could have been introduced due to the difference in population size of the regions in Ethiopia. Moreover, a multilevel, order logistic regression was done to account for any regional variation in the levels of HIV-related knowledge across the regions of the country.

### Limitation of the study

Limitations of this study include the use of measurement questions, for instance, the domains used for measuring HIV knowledge and other important variables, which are limited to variables in EDHS 2016 only. As the current study used data from a single cross-sectional survey, the temporality between HIV knowledge and the factors included in the study cannot be ascertained and the shreds of evidence should be utilized with care. On the other hand, since the 2016 EDHS, which was used for the current study is lacking qualitative data, the authors were unable to explore the link between socio-cultural factors and HIV-related knowledge among the youths.

## Conclusion

Regardless of all efforts put in place to prevent HIV/AIDS in Ethiopia since its first footstep in the country, still less than one-third (30.31%) of Ethiopian youths have deep insight on the disease, whereas, nearly one-fifth of the youths have a lower level of knowledge on the disease. This study has also evidenced that there are significant disparities in HIV-related knowledge among Ethiopian youths living in different regions of the country. Rural residents, less educated, female, and married youths have less knowledge of HIV as compared to their counterparts. On the other hand, youths who do not have a mobile phone, who have no health insurance coverage, and who have limited access to media have less knowledge of HIV. Ethiopia is committed to ending AIDS as a public health threat by 2030. But achieving this goal without scaling up the current lower level of HIV-knowledge among the youths of the country is super absurd and futile efforts. Therefore, any stakeholder working on HIV prevention and control should give due focus to promoting education among the country’s youths by integrating and mainstreaming HIV-related education in the academic curricula, promoting HIV-related awareness creation through community-based education and religious organizations, encouraging and empowering female and rural resident youths, promoting health insurance, and expanding media coverages to best realize their mission.

## Data Availability

All data and materials used in this study are openly accessed and available on a public domain MEASUREDHS website and its accessing link is displayed below. https://dhsprogram.com/data/dataset_admin/login_main.cfm?CFID=10106966&CFTOKEN=a531226989613ac0-7B7AD8A7-E45D-2B2E-C20F5CFFAB6B0B60

## Abbreviations

AIC: Akaike Information Criterion
AIDS: Acquired immune deficiency syndrome
AOR: Adjusted Odds Ration
ART: Anti-Retroviral Therapy
BIC: Bayesian Information Criterion
CI: Confidence Interval
CSA: Central Statistical Agency
DHS: Demographic and Health Survey
EA: Enumeration Area
EDHS: Ethiopian
HIV: Human Immuno Virus
ICC: Intraclass correlation
MoH: Ministry of Health
PLWH: People living with HIV
PHC: Population and Housing Census
PPS: Probability Proportionate to Size
SSA: Sub Saharan Africa
STIs: Sexually Transmitted Infections
UNAIDS: United Nations Program on HIV/AIDS
USAID: United States Agency for International Development
VCT: Voluntary Counseling and Testing
WHO: World Health Organization
